# Semantic Verbal Fluency in Children with and without Autism Spectrum Disorder: Relationship with Chronological Age and IQ

**DOI:** 10.3389/fpsyg.2016.00921

**Published:** 2016-06-17

**Authors:** Gemma Pastor-Cerezuela, Maria-Inmaculada Fernández-Andrés, Mireia Feo-Álvarez, Francisco González-Sala

**Affiliations:** ^1^Basic Psychology Department, Faculty of Psychology, University of Valencia, ValenciaSpain; ^2^Developmental and Educational Psychology Department, Faculty of Psychology, University of Valencia, ValenciaSpain; ^3^Speech Therapy Service, Manises Hospital, ValenciaSpain

**Keywords:** Autism Spectrum Disorder, clustering, flexibility, fluency, generativity, semantic, switching

## Abstract

We administered a semantic verbal fluency (SVF) task to two groups of children (age range from 5 to 8): 47 diagnosed with Autism Spectrum Disorder (ASD Group) and 53 with typical development (Comparison Group), matched on gender, chronological age, and non-verbal IQ. Four specific indexes were calculated from the SVF task, reflecting the different underlying cognitive strategies used: clustering (component of generativity and lexical-semantic access), and switching (executive component, cognitive flexibility). First, we compared the performance of the two groups on the different SVF task indicators, with the ASD group scoring lower than the Comparison Group, although the difference was greater on switching than on clustering. Second, we analyzed the relationships between the different SVF measures and chronological age, verbal IQ and non-verbal IQ. While in the Comparison Group chronological age was the main predictor of performance on the SVF task, in the ASD Group verbal IQ was the best predictor. In the children with ASD, therefore, greater linguistic competence would be associated with better performance on the SVF task, which should be taken into account in speech therapies designed to achieve improvements in linguistic generativity and cognitive flexibility.

## Introduction

The DSM-5 (American Psychiatric Association [APA], 2013) considers Autism Spectrum Disorder (ASD) to be a neurodevelopmental disorder characterized by: (1) persistent deficits in social communication and social interaction across multiple contexts, and (2) the presence of restricted, repetitive patterns of behavior, interests or activities. The severity of these two criteria determines the severity of the disorder, which can be classified into three levels (1): “Requiring support,” (2): “Requiring substantial support,” and (3): “Requiring very substantial support”). Moreover, symptoms must be present in the early developmental period, they must cause clinically significant impairment in important areas of current functioning, and they must not be better explained by intellectual disabilities. Finally, the ASD diagnosis requires specifying whether there is another associated condition or disorder, such as a language impairment. Deficits in verbal language -which are considered under diagnostic criterion 1- and the heterogeneity and variability of linguistic abilities in children with ASD have become widely recognized and researched in recent years (Groen et al., 2008; Eigsti et al., 2011; Boucher, 2012).

In addition, one of the theoretical frameworks proposed to explain the disorder is the theory of executive dysfunction (Ozonoff, 1997; Hill, 2004). According to this theory, people with ASD would present a deficit in the executive functions, understood as a set of cognitive activities in charge of anticipating and setting goals, making plans and programs, beginning activities and mental operations, time organization and sequencing, comparison, classification and categorization, self-regulation of tasks, and the ability to efficiently carry them out (Lezak et al., 2004). One of the cognitive activities that make up the executive functioning (EF) is generativity, or the ability to generate novel responses, which has often been examined using verbal fluency (VF) tasks. VF is defined as the capacity to produce spontaneous verbal responses without excessive pauses or errors in searching for words (Butman et al., 2000). VF tasks are linguistic production tasks that require the subject to generate words beginning with a particular letter (lexical or phonemic tasks), or words that are exemplars of a certain category (semantic tasks), within a specific time limit. These tasks require the activation of mechanisms to access the lexis stored in the semantic long-term memory, linguistic generativity, and other cognitive skills such as focused attention and verbal short-term memory, sustained attention, organization, monitoring, inhibition of inappropriate responses, cognitive flexibility, strategic search and processing speed.

Specifically, the semantic verbal fluency tasks (SVF) consist of asking the subject to produce the greatest possible number of words pertaining to a certain semantic category (e.g., animals, fruits, jobs, kitchen utensils…) within a certain time limit, usually 60 s. Although performance of these types of tasks has usually been evaluated based on the total number of correct words produced within the established time period, some studies have also used specific indicators of the cognitive strategies used to complete these fluency tasks successfully (Kosmidis et al., 2004). Thus, when generating words on a SVF task, healthy participants produce semantically related words, and then, once a semantic subcategory is exhausted, switch to another subcategory (Troyer et al., 1998). The “switch” to a new subcategory within the participant’s current semantic category is a more efficient strategy than using time and mental resources to try to recover words from the participant’s current subcategory, where he/she has probably already used up his/her lexical repertoire. Thus, two underlying abilities or mental components govern VF performance (Troyer et al., 1997): (1) Clustering -the ability to produce a set of words within a particular subcategory- is the generativity component (Turner, 1999), which reflects the semantic organization of stored memory; and (2) Switching -the ability to shift from one subcategory to another- is an executive component that is responsible for strategic search, response initiation, monitoring, shifting and flexibility (Hurks et al., 2010), and it contributes to maximizing performance. Switching -which is thought to be a relatively effortful process- is a more active strategy than clustering, which is considered a relatively automatic process. These are, then, two differentiated cognitive mechanisms or components, so that in the response the subject produces on a SVF task, there can be an activation of both components sequentially or only one of them (either of the two).

Most studies using fluency tests have been carried out in adults, showing a strong relationship between performance on these tasks and educational (Ardila et al., 2000) and vocabulary level (Ruff et al., 1997). Studies using children have found a direct relationship between performance on these tasks and age (Koren et al., 2005; Kavé et al., 2008), especially between 6 and 12 years old (Korkman et al., 2001; Brocki and Bohlin, 2004; Matute et al., 2004; García et al., 2012), although it is not yet clear at what age performance on these tests reaches adult levels. Specifically, this increase in the total production in these age groups would be related to the use of a greater number of clusters (or subcategories) and making more jumps between subcategories (switches), given that there would be an increase in the capacity to change or jump from one subcategory to another (switching), while the clustering component (the number of words produced within the same subcategory) would remain stable (Sauzéon et al., 2004; Koren et al., 2005; Nieto et al., 2008).

In the case of children with ASD, most studies have only used the overall indicator of VF, that is, the total number of correct words produced, finding worse performance than that of children with typical development in some cases (Verté et al., 2006; Mashal and Kasirer, 2012; Ortiz et al., 2013; Czermainski et al., 2014), but not in others (Boucher, 1988; Dunn et al., 1996; Happé et al., 2006; Corbett et al., 2009; Robinson et al., 2009). Very few studies in people with ASD have used specific indicators of clustering and switching. Begeer et al. (2014) hypothesized that, due to the repetitive and stereotypical patterns of behavior of individuals with ASD and their preference for closed systems (Baron-Cohen and Wheelwright, 2004), they could present a tendency to form larger clusters and stay within a cluster, persevering in a specific subcategory. Thus, they would present a lower number of switches and, therefore, clusters, compared to individuals without ASD. Therefore, these authors expected to obtain larger cluster sizes (clustering) and fewer switches (switching) in the ASD Group. In studies carried out with adolescents and adults (Inokuchi and Kamio, 2013) or only with adults (Spek et al., 2009), no differences were found between individuals with ASD and those with typical development on these specific indicators, although there were differences in the total number of correct words produced. By contrast, in the case of children with ASD, the study by Begeer et al. (2014), carried out with children and adolescents, did not find differences between the ASD group and the comparison group on the total number of correct words produced or on the average cluster size (clustering), although there were fewer switches (switching) in the ASD group.

Objectives and hypotheses of the present study aimed to:

(1) Compare the performance of a group of children with ASD (ASD Group) to that of a group of children with typical development (Comparison Group) -matched on gender, chronological age and non-verbal IQ with the ASD Group- on an SVF task, both for the total number of correct words produced and for the specific indicators of clustering and switching. The performance on the different SVF task indicators evaluated would be expected to be lower in the ASD Group than in the Comparison Group, given the limited linguistic competence and the supposed EF impairment in the ASD group. However, we expect these differences to be greater on switching than on clustering, in agreement with the prediction made by Begeer et al. (2014) about the repetitive and stereotypical patterns of behavior of individuals with ASD and their possible preference for closed systems.
(2) Analyze, in each group of children separately, the relationship between the SVF measures and chronological age, verbal IQ and non-verbal IQ. A significant relationship would be expected between the performance on the different indicators obtained on the SVF task and chronological age and verbal IQ, given that VF is a skill that includes executive and linguistic components. We think these components would improve with the increase in the child’s linguistic competence that takes place due to the maturational processes associated with age and learning processes. We expect the correlations with age to be greater in the case of switching than in clustering, as in the results obtained by Sauzéon et al. (2004), Koren et al. (2005), and Nieto et al. (2008), in accordance with the idea that with age there would be an increase in the capacity to change or jump from one subcategory to another (switching), while the clustering component (the number of words produced within the same subcategory) would remain stable.

## Materials and Methods

### Participants

In the present study, participants consisted of a total of 100 children, with ages ranging from 5 to 8 years old, and non-verbal IQ ranging from 75 to 135 on the Raven test (Raven, 1996). The 100 children were divided into two groups: The ASD Group (*n* = 47) was composed of 40 males and 7 females with a mean age of 80.06 months (*SD* = 13.65), a mean non-verbal IQ of 98.89 (*SD* = 19.52), and a mean verbal IQ of 68.83 (*SD* = 19.19) on the Peabody test (Dunn et al., 2006). The Comparison Group (*n* = 53) was composed of 43 males and 10 females with a mean age of 80.90 months (*SD* = 12.95), a mean non-verbal IQ of 99.64 (*SD* = 16.76), and a mean verbal IQ of 96.00 (*SD* = 14.98) on the Peabody test. Children in the ASD Group had a clinical diagnosis of ASD, according to the criteria of the DSM-IV-TR (American Psychiatric Association [APA], 2000), and they met the diagnostic criteria for level 2 of the DSM-5 (American Psychiatric Association [APA], 2013). They were diagnosed by neuropediatric services from different hospitals in the national health system. These neuropediatric services were responsible for checking compliance with these diagnostic criteria. They referred the children who met the diagnostic criteria to early care units, where the diagnosis was confirmed using more specific instruments, such as the Autism Diagnostic Observation Schedule (ADOS), which was applied by specialized psychologists who had the official accreditation to use this instrument. Moreover, all of them obtained an Autism Index score ≥85 on the Gilliam Autism Rating Scale, Second Edition (GARS-2), indicating a high likelihood of the disorder (Gilliam, 2006). The scores ranged from 85 to 135 (*M* = 99.80, *SD* = 11.30). The children in the ASD Group were attending schools with specific classrooms in which the Treatment and Education of Autistic and Related Communication Handicapped Children (TEACCH) methodology was carried out. These are integrated classrooms included in regular state schools in Valencia (Spain), where students with disorders affecting language and communication are enrolled. The children in the Comparison Group were children with typical development, without any clinical diagnosis, who attended the same schools as the ASD Group, but in the regular modality.

Initially, the ASD Group was composed of a total of 67 children from the 18 schools that voluntarily agreed to participate, but 20 children were excluded from the study for different reasons, such as not being able to understand the Raven test, not having oral language, or not receiving their parents’ informed consent. The Comparison Group was initially made up of 350 children who attended 11 of the 18 schools where the children with ASD were enrolled. The two groups of children were matched one-to-one on non-verbal IQ, chronological age and gender, so that of the initial 350 children without ASD, 53 were selected.

No statistically significant differences were found between the two groups of children on gender (χ^2^ = 0.279, *p* = 0.597, η = *0*.053), chronological age [*F*_(1,98)_ = 0.006, *p* = 0.939, $\eta_{p}^{2}$ = 0.000], or non-verbal IQ [*F*_(1,98)_ = 0.042, *p = 0*.837, $\eta_{p}^{2}$ = 0.000]. Nonetheless, statistically significant differences were found on verbal IQ [*F*_(1,98)_ = 63.001, *p* = 0.000, $\eta_{p}^{2}$ = 0.391], which was higher in the Comparison Group than in the ASD Group.

### Ethics Statement

This study is part of a broader investigation that was approved and funded by the University of Valencia and had the official and written authorization of the General Direction and School Management (Valencia Education, Training and Employment Department). All of the Valencian state schools with TEACCH integrated classrooms were invited, via an informative meeting, to participate in the research. From the schools that voluntarily agreed to participate, some classrooms of 5- to 8-year-old children were selected. The parents of the children gave written informed consent to participate in the research.

### Procedures

Each child’s non-verbal IQ, verbal IQ and SVF task performance were individually evaluated by the school psychologist in a noise- and distraction-free office. In all cases, the tasks were administered on different days and in the same order (first: non-verbal IQ task, second: verbal IQ task, and third: SVF task). Information about autism symptoms was obtained from the GARS-2, by means of an interview with the parents of the ASD Group.

### Measures

#### Raven’s Colored Progressive Matrices (CPM; Raven, 1996)

This is a non-verbal test administered to children between 4 and 9 years old. It is a measure of reasoning ability that provides an estimation of the deductive capacity and the “*g*” factor of general intelligence, which is the ability to solve problems without relying on previous knowledge. It has often been used to match children with intellectual disability -who often have limited language comprehension and expression- to children with typical development in research studies. It contains 36 elements, and the child must choose missing pieces from a series of 6 to 8 elements. We used the non-verbal IQ score provided by the test.

#### Peabody Picture Vocabulary Test, (PPVT-III; Dunn et al., 2006)

This instrument is widely used to assess receptive vocabulary. It consists of 192 items: the examiner names a word (noun, verb, adjective, etc.), and the child has to point out an image from four images presented. This implies a decision making process, in which EF is also implicated. We chose this instrument in order to estimate the linguistic competence level in the children of our sample, given that it does not require an oral or written response, and it is a test that can be rapidly administered and utilized to evaluate people with language problems. Thus, according to Bell et al. (2001), the Peabody test should only be used instead of others -such as the Wechsler verbal scales- in individuals with articulation or expressive language problems who cannot be assessed with the Wechsler verbal subtests. This was our case with the group of children with ASD. We used the verbal IQ score provided by this test. In some previous studies, the validity of the Peabody test is evidenced by strong correlations between the Peabody scores and overall intelligence (Bell et al., 2001; Bee and Boyd, 2004), as it is an instrument that has been used in several investigations on ASD in order to obtain an estimation of verbal IQ (e.g., Hala et al., 2007; Pellicano, 2010; Pring et al., 2010; Lam and Yeung, 2012).

#### Gilliam Autism Rating Scale, Second Edition (GARS-2; Gilliam, 2006)

This is a screening scale that provides a norm-referenced measure that helps to identify autism and estimate its severity. It can be filled out by professionals or parents of people between 3 and 22 years old. Based on the DSM-IV-TR diagnostic criteria (American Psychiatric Association [APA], 2000), the scale consists of 42 items that measure three domains associated with the disorder: Stereotyped Behavior, Communication, and Social Interaction. The combined scores on these subscales yield an Autism Index (AI) score (*M* = 100 and *SD* = 15). The higher the value obtained on the global index (AI score), the greater the probability of autism. Depending on the score obtained, three categories are established: *Improbable Autism* (AI score below 70), *Possible Autism* (AI score from 70 to 84), or *Probable Autism* (AI score equal to or greater than 85). Gilliam (2006) reported AI scores ≥85 for 90% of a normative sample of 1,107 people diagnosed with autism. The GARS-2 is a widely-used tool to assess ASD symptoms, and it has been adapted and validated in different countries, with results showing good psychometric characteristics. For the Spanish version, the scale’s internal consistency was high (Cronbach’s alpha = 0.94 for the AI), and the scale’s criterion validity with the Autism Behavior Checklist was also high (0.94).

#### Verbal Expression Subtest of the ITPA (Illinois Test of Psycholinguistic Aptitudes; Kirk et al., 2004)

The task on this ITPA subscale consists of eliciting as many words from a specific semantic category as possible within a time limit of 60 s. It has four different categories: words, body parts, animals, and fruits. For this study, only the semantic category of animals was used, as it is one of the most widely used semantic categories in SVF studies (Lezak et al., 2004). Based on this task, four indices were considered for the study of SVF (Troyer et al., 1997): the total number of correct words produced, the number of changes or switches between groupings of semantic sub-categories (switching, as executive and flexibility indicator), the average size of the clusters (clustering, as an indicator of generativity and lexical-semantic access), and the number of groupings or semantic subcategories (number of clusters), with this latter index used as an additional measure of cognitive flexibility (Raskin et al., 1992).

The definition of the different types of semantic subcategories and the calculation of the indicators was carried out according to the criteria proposed by Robert et al. (1998) and Troyer (2000), complementing it with suggestions from studies conducted with children (Sauzéon et al., 2004; Koren et al., 2005; Nieto et al., 2008). Thus, the total number of correct words was calculated by adding together all the words produced, excluding the errors and repetitions. The number of jumps, changes or switches between groupings or semantic subcategories (or switching) was calculated as the number of transitions between groupings (subcategories or clusters), including isolated words. For example, if the child’s response consists of three semantic subgroupings (or clusters) and four isolated words, six jumps will be counted (or switches). The average size of the groupings, clusters or semantic subcategories (or clustering) was calculated by counting from the second word in a cluster, excluding the isolated words (e.g., two words form a size 1 cluster, three words form a size 2 cluster; four words form a size 3 cluster, and so forth). To calculate the number of groupings or semantic subcategories (number of clusters), a cluster was considered the grouping formed by the successive generation of at least two words within the same semantic subcategory. Therefore, the isolated words are not counted as clusters. The semantic groupings or subcategories (clusters) were: domestic/farm animals, mountain animals, tropical/jungle animals, animals that fly, sea animals, insects, and word pairs (the latter refers to pairs of words that have a strong relationship with each other due to being part of the popular culture or included in fables or tales). If the same semantic subcategory appeared more than once in the child’s response (or cluster), it was counted every time, as the variable is the number of clusters (not the number of different clusters). For example, if a child named domestic animals, then jungle animals, and then domestic animals again, it was coded as three clusters.

Next we present, as an example, a word list generated by one of the participants: cat, cow, chicken, owl, elephant, tiger, lion, leopard, ant, spider, fly, whale, and shark. In this case, the total number of correct words was 13, the number of switches was 4, the number of clusters was 4, and the average size of the clusters was 2. All the calculations were made independently by two of the authors of the manuscript, who were blind to group membership. Inter-rater reliability was calculated for each indicator using Pearson correlation coefficients, with all the correlations above 0.9 and significant at the 0.01 level.

### Data Analysis

Analyses were performed with the SPSS statistical package, version 19 for Windows. First, multivariate analyses of variance (MANOVA) were carried out to compare the SVF measures for the ASD Group and the Comparison Group. Second, for each group separately, Pearson correlation analyses were conducted of the SVF measures and chronological age, non-verbal IQ and verbal IQ. The results of these analyses suggest that the key variables were chronological age and verbal IQ; therefore, we performed Pearson correlation analyses to study the relationship between them in each group. Additionally, in order to investigate whether there was any association between the severity of the autism symptomatology and the SVF performance, Pearson correlation analyses were conducted of autism severity (the global index or AI score, obtained from the Gars-2) and the SVF measures in the ASD Group. Finally, to investigate whether these factors contributed significantly to the explained variance of the SVF measures, in each group separately (ASD Group and Comparison Group), we performed several hierarchical regression analyses, one for each SVF measure. In the case of the ASD Group, the SVF measure correlations are only statistically significant with verbal IQ, but not with the other variables. For this reason, non-verbal IQ, chronological age and gender were entered as covariates in the first step, and then verbal IQ was entered as a predictor variable in the second step, in order to find out the percentage of variance of each of the SVF measures explained by verbal IQ. In the case of the Comparison Group, the SVF measure correlations were only statistically significant with chronological age, but not with the other variables. Therefore, non-verbal IQ, verbal IQ, and gender were entered as covariates in the first step, and then chronological age was entered as a predictor variable in the second step, in order to find out the percentage of variance of each of the SVF measures explained by chronological age.

## Results

### Group Differences in SVF

The MANOVA performed with the scores obtained on the SVF measures revealed statistically significant differences between the ASD Group and the Comparison Group [Wilk’s Lambda (λ) = 0.82; *F*_(4,95)_ = 5.01; *p* = 0.001; $\eta_{p}^{2}$ = 0.174]. As shown in **Table 1**, on all the SVF measures, the Comparison Group obtained higher scores than the ASD Group. The greatest differences were obtained on the measures of total correct words produced, number of clusters, and number of switches (switching), while in the case of the average size of the clusters (clustering), there was less difference between the two groups.

**Table 1:** Means, standard deviations, and *F*-values for the semantic verbal fluency measures for ASD and comparison groups.

	ASD		Comparison		*F*_(1,98)_	*p*	*η^2^*
	*M*	*SD*	*M*	*SD*			
Total correct words	5.98	4.28	9.17	3.25	17.83**	0.000	0.154
Number of clusters	1.55	1.29	2.47	1.18	13.66**	0.000	0.122
Average cluster size (or clustering)	1.63	1.74	2.23	1.13	4.23*	0.042	0.041
Number of switches (or switching)	2.57	2.38	4.00	1.74	11.83**	0.001	0.108

*p < 0.05, **p < 0.01.

### SVF and Chronological Age

Pearson correlations were carried out to examine the relationship between the SVF measures and chronological age in the two groups separately. Regarding the ASD group, there were no statistically significant correlations. Regarding the Comparison Group, all the SVF measures -with the exception of the average cluster size, or clustering- showed a statistically significant correlation with chronological age (**Table 2**). **Tables 3** and **4** present the results of the hierarchical regression analysis conducted in the Comparison Group to calculate the explained variance for the SVF measures. The gender, verbal IQ and non-verbal IQ variables explained a very low percentage of the variance in the SVF measures. However, chronological age explained statistically significant percentages of variance in all the SVF measures (with the exception of the average cluster sizes, or clustering): 35.7% in number of clusters, 22.4% in total correct words produced, and 11.8% in number of switches (or switching).

**Table 2:** Correlations between the semantic verbal fluency measures (total correct words, number of clusters, average cluster size, and number of switches) and chronological age (CA), non-verbal IQ (NVIQ), and verbal IQ (VIQ), in ASD Group and Comparison Group.

	ASD				Comparison		
		CA	NVIQ	VIQ	CA	NVIQ	VIQ
Total correct words	*r*	-0.081	0.083	0.519**	0.329*	0.148	0.162
	*p*	0.589	0.578	0.000	0.016	0.289	0.246
Number of clusters	*r*	-0.276	0.158	0.407**	0.502**	0.009	0.053
	*p*	0.060	0.290	0.005	0.000	0.951	0.706
Average cluster size (or clustering)	*r*	-0.106	0.224	0.357*	-0.179	0.157	0.199
	*p*	0.479	0.131	0.014	0.199	0.260	0.153
Number of switches (or switching)	*r*	0.068	-0.046	0.408**	0.285*	0.081	-0.007
	*p*	0.651	0.757	0.004	0.038	0.564	0.958

**p < 0.01, *p < 0.05.

**Table 3:** Hierarchical regression analyses for gender, CA, NVIQ, and VIQ predicting the semantic verbal fluency measures in the Comparison Group.

Variables	*R*^2^	Adjusted *R*^2^	*B*	β	*F* Change
**Total correct words**					
Step 1					
Gender, VIQ, NVIQ	0.03	0.03			0.61
Step 2					
Gender, VIQ, NVIQ	0.03	0.03			0.61
CA	0.26	0.22	0.13	0.55**	0.00**
**Number of clusters**					
Step 1					
Gender, VIQ, NVIQ	0.00	0.00			0.98
Step 2					
Gender, VIQ, NVIQ	0.00	0.00			0.98
CA	0.36	0.35	0.06	0.69**	0.00**
**Average cluster size**					
Step 1					
Gender, VIQ, NVIQ	0.04	0.04			0.51
Step 2					
Gender, VIQ, NVIQ	0.04	0.04			0.51
CA	0.05	0.01	-0.01	-0.11	0.48
**Number of switches**					
Step 1					
Gender, VIQ, NVIQ	0.06	0.06			0.34
Step 2					
Gender, VIQ, NVIQ	0.06	0.06			0.34
CA	0.18	0.11	0.05	0.39*	0.01*

**p < 0.01, *p < 0.05.

**Table 4:** Coefficients of the variables in the regression models from **Table 3** (Comparison Group).

	Step 1				Step 2			
Variables	*B*	*SE*	β	*t*	*B*	*SE*	β	*t*
**Total correct words**								
Constant	4.18	3.79		1.10	-12.30	5.47		-2.24*
Gender	0.44	1.18	0.05	0.38	-0.03	1.05	-.00	-0.03
VIQ	0.01	0.031	0.08	0.51	0.06	0.03	0.33	2.12*
NVIQ	0.02	0.034	0.12	0.78	0.04	0.03	0.18	1.31
CA					0.13	0.03	0.55	3.81**
**Number of clusters**								
Constant	2.17	1.40		1.55	-5.39	1.85		-2.90**
Gender	-0.02	0.43	-0.01	-0.06	-0.24	0.35	-.08	-0.69
VIQ	-0.00	0.01	-0.02	-0.11	0.02	0.01	0.30	2.05*
NVIQ	0.00	0.01	0.06	0.38	0.01	0.01	0.13	1.04
CA					0.06	0.01	0.69	5.17**
**Average cluster size**								
Constant	0.57	1.32		0.43	1.77	2.16		0.82
Gender	-0.05	0.41	-0.02	-0.14	-0.02	0.41	-0.00	-0.05
VIQ	0.00	0.01	0.09	0.55	0.00	0.01	0.03	0.20
NVIQ	0.01	0.01	0.16	1.01	0.01	0.01	0.14	0.91
CA					-0.01	0.01	-0.11	-0.70
**Number of switches**								
Constant	1.90	2.00		0.95	-4.49	3.07		-1.46
Gender	1.06	0.62	0.24	1.71	0.88	0.59	0.20	1.49
VIQ	0.00	0.01	0.05	0.31	0.02	0.01	0.23	1.41
NVIQ	-0.00	0.01	-0.03	-0.20	0.00	0.01	0.01	0.08
CA					0.05	0.02	0.39	2.63*

**p < 0.01, *p < 0.05.

### SVF and Verbal IQ

Pearson correlations were carried out to examine the relationship between the SVF measures and verbal IQ in the two groups separately. Regarding the ASD group, all the SVF measures showed a statistically significant correlation with verbal IQ. Regarding the Comparison Group, there were no statistically significant correlations (**Table 2**). **Tables 5** and **6** present the results of the hierarchical regression analysis conducted in the ASD Group to calculate the explained variance for the VF measures. The gender, chronological age and non-verbal IQ variables explained a very low percentage of variance in the SVF measures. However, verbal IQ explained statistically significant percentages of variance in all the SVF measures: 30.8% in total correct words produced, 26.1% in number of switches (or switching), 12.2% in number of clusters, and 8.6% in average cluster size (or clustering).

**Table 5:** Hierarchical regression analyses for gender, CA, NVIQ, and VIQ predicting the semantic verbal fluency measures in the ASD Group.

Variables	*R*^2^	Δ*R*^2^	*B*	β	*F* Change
**Total correct words**					
Step 1					
Gender, CA, NVIQ	0.01	0.01			0.93
Step 2					
Gender, CA, NVIQ	0.01	0.01			0.93
VIQ	0.31	0.30	0.14	0.65**	0.00**
**Number of clusters**					
Step 1					
Gender, CA, NVIQ	0.09	0.09			0.24
Step 2					
Gender, CA, NVIQ	0.09	0.09			0.24
VIQ	0.21	0.12	0.02	0.41*	0.01*
**Average cluster size**					
Step 1					
Gender, CA, NVIQ	0.06	0.06			0.44
Step 2					
Gender, CA, NVIQ	0.06	0.06			0.44
VIQ	0.14	0.08	0.03	0.34*	0.04*
**Number of switches**					
Step 1					
Gender, CA, NVIQ	0.00	0.00			0.94
Step 2					
Gender, CA, NVIQ	0.00	0.00			0.94
VIQ	0.26	0.26	0.07	0.60**	0.00**

**p < 0.01, *p < 0.05.

**Table 6:** Coefficients of the variables in the regression models from **Table 5** (ASD Group).

	Step 1				Step 2			
Variables	*B*	*SE*	β	*t*	*B*	*SE*	β	*t*
**Total correct words**								
Constant	6.43	6.95		0.92	0.96	5.97		0.16
Gender	-0.01	0.05	-0.04	-0.28	-0.00	0.04	-0.00	-0.02
CA	-0.32	1.85	-0.02	-0.17	0.37	1.56	0.03	0.24
NVIQ	0.01	0.03	0.06	0.38	-0.05	0.03	-0.26	-1.62
VIQ					0.14	0.03	0.65	4.35**
**Number of clusters**								
Constant	3.62	2.02		1.79	2.57	1.94		1.32
Gender	-0.02	0.01	-0.23	-1.39	-0.02	0.02	-0.20	-1.28
CA	-0.41	0.54	-0.11	-0.76	-0.28	0.51	-0.07	-0.54
NVIQ	0.01	0.01	0.07	0.43	-0.01	0.01	-0.13	-0.78
VIQ					0.03	0.01	0.41	2.55*
**Average cluster size**								
Constant	-0.66	2.758		-0.23	-1.84	2.72		-0.67
Gender	-0.00	0.02	-0.03	-0.22	-0.00	0.02	-0.01	-0.08
CA	0.49	0.73	0.10	0.67	0.64	0.71	0.13	0.90
NVIQ	0.02	0.01	0.20	1.22	0.00	0.02	0.03	0.15
VIQ					0.03	0.01	0.35	2.06*
**Number of switches**								
Constant	2.42	3.87		0.62	-0.38	3.445		-0.11
Gender	0.01	0.03	0.07	0.42	0.02	0.02	0.11	0.77
CA	-0.40	1.03	-0.06	-0.39	-0.04	0.90	-0.00	-0.05
NVIQ	-0.00	0.02	-0.01	-0.06	-0.03	0.02	-0.31	-1.86
VIQ					0.07	0.02	0.60	3.87**

**p < 0.01, *p < 0.05.

### SVF and Non-verbal IQ

Pearson correlations were carried out to examine the relationship between the SVF measures and non-verbal IQ in the two groups separately. There were no statistically significant correlations in either of the two groups (**Table 2**).

### Chronological Age and Verbal IQ

Pearson correlations were carried out to examine the relationship between chronological age and verbal IQ in the two groups separately. Regarding the Comparison Group, there was a statistically significant correlation (*r* = -0.320, *p* = 0.019), indicating an inverse relationship between age and verbal IQ in the children in the Comparison Group of our sample. Regarding the ASD Group, an inverse relationship was also obtained between the two variables, in this case with an almost marginal significance level (*r* = -0.294, *p* = 0.045).

### SVF and Autism Severity

Pearson correlations were carried out to examine the relationship between the SVF measures and autism severity in the ASD Group. We did not find associations between autism severity and any of the measures from the SVF task (*r* = -0.088, *p* = 0.556 on the total number of correct words; *r* = 0.009, *p* = 0.952 on the number of clusters; *r* = -0.110, *p* = 0.461 on the average size of clusters; and *r* = -0.109, *p* = 0.464 on the number of switches).

## Discussion

Verbal fluency tasks are linguistic production tasks that are considered a good indicator of EF (Henry and Crawford, 2004) and have been linked to prefrontal cortex activation (Lezak et al., 2004). Specifically, SVF tasks require the subject to generate words that are exemplars of a certain category within a specific time limit. These types of tasks have been more specifically linked to temporal cortex activation (Newcombe, 1969; Martin et al., 1990; Baldo et al., 2006). SVF tasks require linguistic generativity skills and semantic-lexical access, as well as executive skills such as cognitive flexibility, strategy search, inhibition and set shifting, skills that can be impaired in individuals with ASD (Lopez et al., 2005; Russo et al., 2007). In the case of the linguistic skills, the possible presence of limitations or impairments in verbal language is an aspect that needs to be specified as part of the diagnostic criteria for the disorder (American Psychiatric Association [APA], 2013). The majority of the studies that have used VF tasks in people with ASD have only considered the overall measure of the number of correct words produced. Therefore, it is not possible to know which underlying cognitive strategies are used in performing these types of tasks. The few studies that have analyzed the specific indicators of VF -clustering and switching- in individuals with ASD have used very high functioning clinical groups with strong linguistic skills and broad age ranges (Spek et al., 2009; Begeer et al., 2014). To the best of our knowledge, our study is the first one to analyze the specific indicators of VF -clustering and switching- in a clinical sample of children with ASD, with a limited age range (from 5 to 8 years old) and with limited linguistic skills (the verbal IQ mean was around 69), placing them at level 2 of ASD severity, according to the DSM-5 classification (American Psychiatric Association [APA], 2013). We used a comparison group of children without ASD, matched one-to-one with the children in the ASD Group on gender, chronological age and non-verbal IQ.

The first objective of our study was to compare the performance of the two groups of children on the different specific indicators of the SVF task. As expected, on all the SVF measures, the ASD Group performed worse than the Comparison Group, showing more limited skills on linguistic generativity and cognitive flexibility. The children in the ASD Group produced a lower total number of correct words than the children in the Comparison Group, and they also made fewer jumps, changes or switches from one semantic subcategory to another (switching) and, therefore, obtained fewer clusters. Regarding the average cluster size (or clustering), the difference between the two groups, although reaching statistical significance, was much smaller than in the case of the other measures. Therefore, although there were differences between the two groups in the relatively automatic process of recovering information stored in the semantic long-term memory and producing words, the differences were much greater in the processes of strategic search, cognitive flexibility and set shifting, which, in a more controlled and conscious way, are necessary when performing a SVF task. In summary, switching -as the executive component involved in the task- would be especially affected, in agreement with the theory of executive dysfunction in ASD (Ozonoff, 1997; Hill, 2004).

On some of the SVF task indicators, the results we obtained would agree with the Begeer et al. (2014) hypothesis. This hypothesis states that the tendency of people with ASD toward perseveration and systematization (Baron-Cohen and Wheelwright, 2004) would lead them to form larger clusters and stay within a cluster (that is, a high average cluster size, or clustering). This tendency would then lead them to make a low number of jumps, changes or switches to other subcategories or clusters (that is, less switching) and, therefore, have a low number of clusters. The idea that people with ASD present a tendency toward perseveration and systematization would be directly related to the hyper-selectivity (or a detail-focused style of processing) proposed within the framework of the Weak Central Coherence Theory (Frith and Happé, 1994) and the Enhanced Perceptual Functioning Theory (Mottron and Burack, 2001). The latter offers a positive view of the differences in information processing in ASD – compared to typical development –, by proposing an enhanced local processing and a higher perception of details in people with ASD. On the SVF task, this hyper-selectivity -or tendency toward a narrow attentional focus- in the ASD people would be associated with a tendency to remain within the same cluster, producing a greater number of words within it and a lower tendency to jump or change cluster (and, therefore, generating a lower number of clusters), in comparison to people with typical development. In our results, we obtained a lower number of jumps and a lower number of clusters in the ASD group than in the Comparison Group. However, neither our study nor the one by Begeer et al. (2014) was able to confirm that the clustering (the average size of the clusters) was greater in the ASD group than in the Comparison Group. In any case, given that at the ages considered in our study the children are in the process of developing their executive and linguistic skills, it would be interesting to follow these children over time through a longitudinal study to find out whether the differences between the two groups in the specific SVF task indicators change or are maintained over time.

The second objective of our study was to analyze, in each group separately, the relationships of the different specific SVF indicators with chronological age, verbal IQ and non-verbal IQ. In the case of the Comparison Group, we obtained significant correlations between age and the measures of the total number of correct words produced, the number of clusters, and the number of switches (or switching), but not with the average cluster size (or clustering). These results would support the idea that, at the ages considered, there would be an increase in the total production, the use of a greater number of clusters, and more jumps (or switches) made, given that there would be an increase in the capacity to change or jump from one subcategory to another (switching), while the clustering component (the number of words in the same subcategory) would remain stable (Sauzéon et al., 2004; Koren et al., 2005; Nieto et al., 2008).

Moreover, in the Comparison Group, we did not obtain a relationship between any of the specific SVF indicators and verbal IQ or non-verbal IQ. In the age ranges considered, IQ does not seem to be associated with performance on the SFV task in children with typical development, as revealed in the results of the multiple regression analyses. Chronological age was the only variable that explained a statistically significant percentage of variance in the specific measures of SVF (with the exception of the average cluster size, or clustering). Therefore, in the children with typical development, age was an important variable in predicting the total number of correct words produced, as well as the number of clusters and switches (or switching), but not the size of the clusters (or clustering). However, verbal and non-verbal IQ were not relevant variables in predicting the performance on any of the indicators obtained from the SVF task. With regard to age, the results obtained in this study reinforce those obtained by previous studies in children with typical development, where a direct relationship was found between the performance on VF tasks and age (Koren et al., 2005; Kavé et al., 2008), especially between 6 and 12 years old (Korkman et al., 2001; Brocki and Bohlin, 2004; Matute et al., 2004; García et al., 2012).

Additionally, the results of the analysis of the relationship between chronological age and verbal IQ in the Comparison Group indicated an inverse relationship between the two variables, which would support the idea that the improvement in the performance on the SVF task that is produced with greater chronological age would not be associated with higher verbal IQ. Given that, in addition, the switching component is related to age, but the cluster component is not, we hypothesized that the improvement in the executive component – and not the linguistic one- would be the main factor associated with the SVF task improvement that occurs with age in this group.

In the case of the ASD Group, neither chronological age nor non-verbal IQ correlated with any of the measures obtained on the SVF task. In this group, verbal IQ correlated significantly with all the measures obtained from the SVF task: total number of correct words produced, number of switches (or switching), number of clusters, and average cluster size (or clustering). In the age range considered, neither age nor non-verbal IQ seems to be associated with performance on the SFV task in children with ASD, as the results of the multiple regression analyses revealed. Verbal IQ was the only variable that explained statistically significant percentages of variance in each of the specific measures from the SVF. Therefore, in the children with ASD, only linguistic skills were shown to be an important variable in predicting performance on the SVF task.

In addition, the results of the analysis of the relationship between chronological age and verbal IQ in the ASD Group indicates an inverse relationship- almost marginally significant- between the two variables, which would support the idea that the improvement in the performance on the SVF task that occurs with a higher verbal IQ would not be associated with chronological age. Furthermore, the absence of a relationship between the performance on the SVF task and the severity of the autism symptomatology also supports the idea that the level of linguistic competence or ability would be the main factor associated with the performance on the SVF task in the ASD Group, given that other factors such as age and autism severity were not found to be associated with the task performance in this group.

In summary, while in the group of children with typical development, age was the main predictor of performance on the SVF task, in the case of the children with ASD, age alone was not a significant predictor of performance on the SVF task, but verbal IQ was. Although, we believe it would be advisable to carry out future studies along the same lines in order to verify the reach of these conclusions, the idea seems important that, in children with ASD, greater linguistic competence would be associated with better performance on the SVF task. This leads us to hypothesize that to achieve improvements in children with ASD on linguistic generativity, semantic-lexical access, cognitive flexibility and search strategies- the most relevant skills involved in SVF task performance-, learning processes and the development of linguistic skills through adequate intervention would be more important than maturation due to age. Therefore, speech therapy and verbal language stimulation therapies for children with ASD should be directed not only toward pragmatic aspects (communicative and functional aspects of language), but also toward other aspects of language that are necessary to reach good linguistic competences (e.g., morphological and syntactic aspects). These intervention techniques could contribute to strengthening the necessary cognitive skills to improve the performance of children with ASD on SFV tasks.

In any case, it would be interesting to investigate the mutual influence between executive and language impairments. In this sense, some hypotheses have been proposed, such as the language mediation hypothesis of executive dysfunctions in autism (Russel, 1997), which considers that the executive deficits in this disorder are secondary to primary deficits in the ability to use inner speech to control and guide behaviors (Hughes and Russell, 1993). The possibility that an impairment in language may induce secondary impairment in executive functions has been investigated in some studies (Joseph et al., 2005; Narzisi et al., 2013). However, the results are still not sufficiently conclusive to be able to establish whether a lack of language mediation may explain the executive function deficits or whether language and executive functions are simultaneously impaired in autism.

### Limitations

Our study presents some limitations. First, not all of the autism spectrum disorder was represented because children with serious behavioral problems or very low cognitive functioning were not part of the sample. One of our objectives was to study the relationship between the different indicators of the SVF task and IQ, but it is important to note that there were no participants in this study with a non-verbal IQ under 75. Second, there is no information about whether the children had received or were receiving speech therapy or any other treatments at the time of the evaluation. Third, this research used cross-sectional data, so that it did not study the variables over time. Finally, this research did not include a comparison group with a different psychological disorder -e.g., ADHD-, and so we cannot definitively conclude that the group differences were unique to autism.

## Author Contributions

Conceived and designed the work: M-IF-A, GP-C, and MF-A. Acquired data: M-IF-A, GP-C, and FG-S. Coded data: MF-A and GP-C. Corrected data: MF-A and FG-S. Analyzed data: M-IF-A and GP-C. Interpreted data: M-IF-A, GP-C, MF-A, and FG-S. Wrote the paper: M-IF-A, GP-C, and MF-A. Drafted the article and revised it critically: GP-C and FG-S.

## Conflict of Interest Statement

The authors declare that the research was conducted in the absence of any commercial or financial relationships that could be construed as a potential conflict of interest. The reviewer ZS and handling Editor declared their shared affiliation, and the handling Editor states that the process nevertheless met the standards of a fair and objective review.
